# Predictors of weight reduction in a Nigerian family practice setting

**DOI:** 10.4314/gmj.v56i3.11

**Published:** 2022-09

**Authors:** Adetola M Ogunbode, Mayowa O Owolabi, Olayinka O Ogunbode, Lawrence A Adebusoye, Adesola Ogunniyi

**Affiliations:** 1 Department of Family Medicine, University College Hospital (U.C.H.), Ibadan, Oyo State, Nigeria; 2 Department of Medicine, College of Medicine, University of Ibadan, Ibadan, Oyo State, Nigeria; 3 Department of Obstetrics and Gynaecology, College of Medicine, University of Ibadan, Ibadan, Oyo State, Nigeria

**Keywords:** Predictors, weight reduction, obesity, Patient Information Leaflets, Model

## Abstract

**Objectives:**

This study identified the predictors of weight reduction among adult obese patients in a Family Practice Setting and developed a statistical model to predict weight reduction.

**Design:**

A prospective cohort design.

**Setting:**

The Family Practice Clinic, University College Hospital, Ibadan, Nigeria

**Participants and study tools:**

Obese adults were recruited into a three-month weight reduction program. Patient Information Leaflets were used for counselling, while questionnaires were administered to obtain socio-demographic and lifestyle factors. Potential predictors were assessed using the Multidimensional Scale of Perceived Social Support, Zung Depression Scale, Rosenberg Self-Esteem scale, Garner's Eating Attitude Test-26 (EAT-26), 24-hour dietary recall and International Physical Activity Questionnaire-short form. Anthropometric indices, blood pressure and Fasting Lipid Profile were assessed. Descriptive and inferential statistics were used for analysis with a significance set at α0.05.

**Results:**

Most 99(76.2%) of the 130 participants achieved weight reduction and had a median weight change of -2.3kg (IQR-4, -0.5), with 66 (66.7%) out of 99 attaining the weight reduction target of 10%. The regression model showed predictors of weight reduction to be Total Cholesterol [TC] (p=0.01) and Low-Density Lipoprotein Cholesterol [LDL-C] (p=0.03). The statistical model derived for Weight reduction = 0.0028 (LDL-C) -0.029 (TC)-0.053 (EAT-26) +0.041(High-Density Lipoprotein Cholesterol). The proportion of variance of the model tested was R^2^ = 0.3928 (adjusted R^2^ = 0.2106).

**Conclusion:**

Predictors of weight reduction among patients were eating attitude score, Total Cholesterol, Low-Density Lipid and High-Density Lipoprotein Cholesterol levels. A statistical model was developed for managing obesity among patients.

**Funding:**

Self-funded, with a discount from the Public-Private Partnership Laboratory obtained for the patients recruited.

## Introduction

Obesity, a risk factor for Non-Communicable Diseases, is a global public health concern in both developed and developing countries because of increasing westernisation and the adoption of foreign lifestyles.^1^,^2^ Obesity has several health risks with multi-systemic complications, including type 2 diabetes mellitus, dyslipidaemia, hypertension, increased depression and lower self-esteem.^4^ It is estimated that over one billion adults are overweight, out of which at least 300 million have obesity.^1^ WHO also reported a 50% increase in the number of adults with obesity worldwide between 1995 and 2000.^5^

From the NHANES, 2013–2014, in the U.S.A., obesity was reported to have risen to 38%^6^ from the 30.5% documented in 1999–2000.^5^ In 2015, in the U.K., the obesity rate was 26.2%, and in Turkey, obesity prevalence was documented as 22.3%.^7^ Countries in economic transition from developing to developed, such as China, Brazil and South Africa, are particularly affected and have an increased rate of obesity across all economic levels and age groups. In South Africa, the self-reported prevalence of obesity was documented as 26.5%.^7^

Obesity rates in SSA were documented in 2016 as 10% in rural Uganda, 14 % in peri-urban Uganda, 31 % in Nigeria, 40 % in Tanzania and 54% in South Africa.^8^

Obesity prevalence has a geographical variation in Nigeria, West Africa, with 41.8% in the South-west, 2 47.3% in South-south, 9 33.3% in the East 10 and 8.1% in the North-central region. 11 In Nigeria, even with a poor Gross Domestic Product (GDP) and a high poverty index, there are documented increasing levels of obesity rising from about 20% between 2002 and 2010 12 to 33.3%. 9,12 The healthcare cost of complications of obesity is large,^13^ consisting of direct and indirect costs.^11^

The various factors driving weight reduction in obesity worldwide are non-modifiable factors, including genetic susceptibility, Leptin and gender, 14,15 and modifiable factors that comprise lifestyle variables such as physical activity and diet. Questions (Qs) 3 and 4 raised by the Obesity Expert Panel are critical to the issue of weight reduction. 16 Q1 was: What are the Cerebrovascular Disease (CVD) health benefits associated with weight reduction? Q2 was: What are the health risks of weight reduction relative to CVD, and secondly, are the limits for Body Mass Index (BMI) and Waist Circumference enough for population subcategories? Q3 was: For weight reduction, what dietary plan can be achieved? Q4 was: Is a package for lifestyle consisting of diet, exercise and behaviour therapy enough? Q5 was: What type of patients would benefit from obesity-related surgery?

The efficacy of behavioural weight reduction treatments is well-known among healthcare professionals in developed countries. Predictions of treatment compliance and weight reduction have been widely proven elusive because obesity varies in different subgroups of patients.^17^ This present study is different because it was aimed at determining the predictors of weight reduction among patients with obesity both in a developing country and in an urban setting in Ibadan, South-west Nigeria, as compared to previous research in rural Nigeria, which determined weight change and influencing factors.^18^,^19^ A secondary objective of this study was to develop a statistical model that can predict individual weight reduction. It will be useful in health facilities with a weight loss program^20^ or in community-based programs.^21^

## Methods

The study was designed as a prospective cohort study among adult patients with obesity who were above 18 years attending the General Outpatients' (G.O.P.) clinic, a Family Practice setting in the University College Hospital, Ibadan, Oyo State, South-Western Nigeria.

The study population was adult patients aged 18 years and above who presented consecutively between 14th August 2013 to 5th May 2016.

The sample size was calculated using the formula for a single estimate, 23, with the average mean weight change (0.39kg per year) among adult Nigerians. 24 The inclusion criteria were obesity (BMI ≥ 30kg/m^2^) and informed consent. The exclusion criteria were those with acute illness, pregnant women, diabetes mellitus, physical deformities affecting the spine and limbs, and prescription weight loss medication.

Institutional ethical approval was obtained from the University of Ibadan/ University College Hospital Ethical Committee, and the registration number was NHREC/05/01/2008a.

An interviewer-administered semi-structured questionnaire was designed and used to obtain relevant information such as socio-demographic characteristics, history of obesity, previous treatment for obesity, with family and lifestyle history. Validated instruments used included the Multidimensional Scale of Perceived Social Support (MSPSS), a 12-item tool that measures support with aggregate scores ranging from 7–84. High acuity/support was 69–84, moderate acuity (49–68) and low acuity (12–48). MSPSS has a Cronbach's alpha coefficient of 0.86 to 0.90. 25

Depression was assessed with the Zung Depression Scale (ZDS), a 20-item questionnaire with a maximum score of 80. Severe depression was scored as ≥70, moderate depression (60 - 69), minimal depression (50 – 59), and < 50 is normal. 26 ZDS, when administered in Yoruba (the local dialect) and English languages, has an index reliability of 0.64 – 0.79. 27,28 The Rosenberg Self-Esteem (RSE) scale is a 10-item tool that measures self-esteem. Each item gives a maximum score of 4 points, on a scale of 10 to 40 points, with higher scores reflecting higher self-esteem. RSE has a reliability and validity of 29 and a Cronbach's alpha of 0.87. 25 The Garner's Eating Attitude Test-26 (EAT-26) has 26 items used in a West African population. 30,31 and has a Likert scale with scores above 20 indicating concerns about body weight, body shape and eating. 32

The 24-hour dietary recall assesses diet and validated face-and content, with cultural adaptations in a previous study. 33 Food portion sizes were estimated using household measures or the hand guide. 34 The Total Dietary Assessment (TDA) software was used to analyse the 24-hour dietary recall data of consumed food and drinks.

A human nutritionist/statistician checked this dietary data inside the Nigerian food composition table. 35

Foods and drinks consumed were converted to a weight equivalent and then inserted into the TDA, which then converted it to caloric intake. The International Physical Activity Questionnaire (IPAQ)-short form (IPAQ-SF) was used to assess physical activity. The IPAQ-SF was categorised into inactive/sedentary, minimally active and Health Enhancing Physical Activity (HEPA) active.^36^ The inactive/sedentary group were further re-categorized into the inactive category, while the minimally active and Health Enhancing Physical Activity (HEPA) active group were categorised into the active group.

### Clinic visits and follow-up

The participants were observed over 3 months at enrollment (first visit), one month later (second visit) and two months after (third visit). At the first visit, every participant was formally identified with a unique number and had their details, such as their telephone numbers, addresses, and initials, written in a logbook. The interviewer administered the questionnaire, and anthropometric and blood pressure measurements were done. For the Fasting Lipid Profile tests, the participants were told to fast overnight for 12–14 hours 37 and have their blood samples collected the following day. An EDTA bottle was used to collect the blood samples. Centrifuging of the blood was performed for 5 minutes at 3000 revolutions per minute after collecting the sample by a qualified Laboratory Scientist. If the analysis was not done the same day, the plasma sample was kept overnight in the fridge at 4°C or frozen at -20 o C in plain plastic bottles. The colourimetric method was used for the estimation of Total Cholesterol (TC), High-Density Lipoprotein (HDL)-Cholesterol and Tri-glycerides (TG) using an auto-analyzer Hitachi 902. The cholesterol oxidase phenol 4- amino antipyrine peroxidase (CHOD-PAP) by Abell-Kendall was used to estimate the TC. In contrast, the triglyceride glycerol-3-phosphate oxidase phenol + aminophenazone (TG GPO-PAP) was used to determine the TG. The HDL was measured using the homogeneous enzymatic colourimetric test, while the Low- Density Lipoprotein (LDL) - cholesterol was calculated using the formula:

LDL =Total cholesterol- (TG/5+HDL).

Patient Information Leaflets (PILs) with a lifestyle modification mnemonic: A-Activity increase, B-Behavioral modification, C- Calorie reduction (ABC); were used to counsel the patients for target weight reduction. The participants were informed and counselled about the investigation results during the second and third visits. The anthropometric and blood pressure measurements were performed.

Target weight reduction was noted and used to classify the participants into either target weight reduction or no target weight reduction group. Participants were referred to specialists such as dietitians and physiotherapists for further management as the need arose.

### Data analysis

Data entry and analysis were carried out with the Statistical Package for Social Sciences version 20 (SPSS-20). Target weight reduction was defined as 10% (2.5% in 3 months) of their baseline weight. Based on existing literature, age was classified into less than and above or above 40 years. 38 Descriptive statistics, such as measures of central tendency like means and medians, were first used to analyse the data, and inferential statistics using the Chi-square test were used. The TDA software was used to analyse the 24-hour dietary recall by a statistician/ Human Nutritionist. Multiple linear regression analysis was employed to arrive at the statistical model for weight reduction. The repeated measure of Analysis of Variance (ANOVA) was also carried out. The level of significance was set at 5%.

## Results

The majority of the 130 participants, 108 (83.1%), were 40 years, with a mean age of 48 (± 8.4) years. Table 1 shows that target weight reduction was greater in those less than 40 years 14 (63.6%), in females 57 (52.8%), and in those with greater than secondary school education 53 (51.0%). A greater proportion of the participants who were not married, 15 (75.0%), achieved the target weight reduction, which was statistically significant (p=0.02). Of those who had blue-collar jobs, 65(51.2%) and an income below 18,000naira (the minimum standard wage as at the time of the study in Nigeria), 12 (54.5%) were able to achieve more target weight reduction.

**Table 1:** Association between the socio-demographic profile of participants and target weight reduction

Variables	No target weight reduction (n=64)		Target weight reduction (n=66)		(χ)	p-value
***Age category (years)***						
***< 40***	8	36.4	14	63.6	1.75	0.19
***≥40***	56	51.9	52	48.2		
***Sex***						
***Male***	13	59.1	9	40.9	1.03	0.31
***Female***	51	47.2	57	52.8		
***Marital status***						
***Married***	59	53.6	51	46.4	5.55	*0.02
***Not Married***	5	25.0	15	75.0		
***Educational level***						
***<Secondary***	13	50.0	13	50.0	0.01	0.93
***≥Secondary***	51	49.0	53	51.0		
***+ Occupation***						
***White-collar***	2	66.7	1	33.3	0.37	0.54
***Blue-collar***	62	48.8	65	51.2		
***Monthly income category (Naira)***						
***<18,000***	10	45.5	12	54.5	0.15	0.70
***≥18,000***	54	50.0	54	50.0		
***Religion***						
***Christianity***	48	52.2	44	47.8	1.09	0.30
***Islam***	16	41.1	22	57.9		
***Ethnicity***						
***Yoruba***	52	48.6	55	51.4	0.97	0.76
***Others***	12	52.2	11	47.8		

*p-value significant

+While collar jobs are not manual jobs, including professionals, engineers, sales representatives, registered nurses, and office managers. In contrast, blue-collar jobs are manual jobs such as machine operators, construction workers, assemblers, etc.^3^

**Figure 1:**
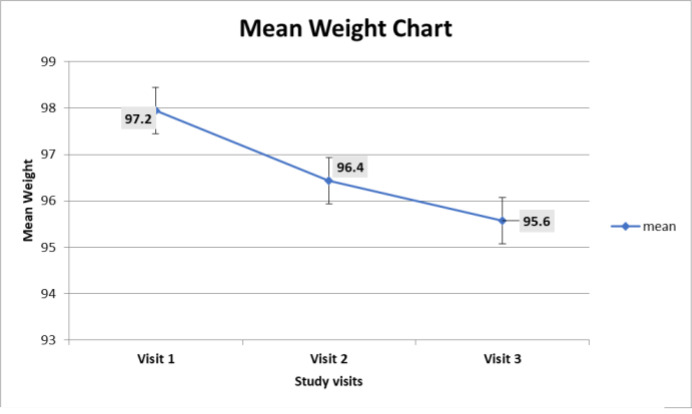
Error-bar chart for mean weight reduction of the participants across the 3 visits

**Figure 2:**
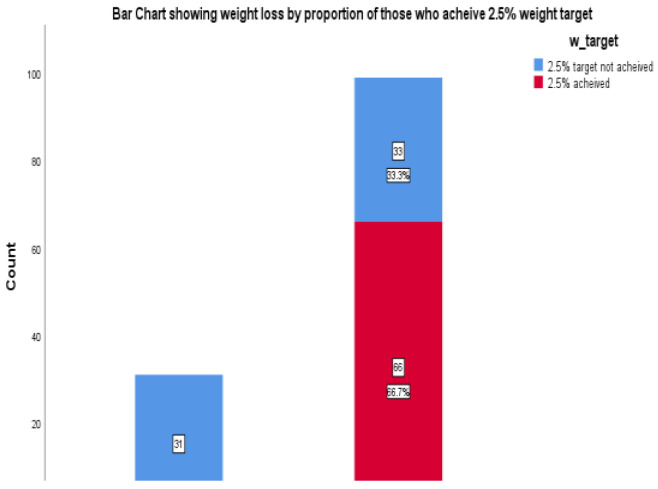
Bar chart for proportion of participants who achieved target weight reduction out of the overall respondents who reduced in weight by the end of the study

Most participants (n=99) achieved a mean weight reduction from 97.2±16.4kg at baseline to 95.6±14.2kg at the end of the study. This is seen in the error-bar chart in Figure 1. The median weight change was -2.3kg (IQR-4, -0.5). Out of all the 130 participants, 99 (76.2%) participants were able to reduce weight by the end of the study, while 66 (66.7%) were able to achieve the target weight reduction of 10% (2.5% in 3 months). This is depicted in Figure 2.

Table 2 shows that half of the participants who achieved target weight reduction were informed about obesity when they were less than 40 years 49 (56.3%). Less than half of the participants who achieved target weight by weight reduction methods tried before 48 (51.6%) had tried to lose weight through exercise-related techniques before the study 15 (53.6%).

**Table 2:** Association between obesity/ family history of obesity and support among participants with target weight reduction

Obesity/family history and support	No target weight reduction n=64	Target weight reduction n=66	χ^2^	p-value
**Age informed about obesity (years)**				
**<40 (Young adult)**	38(43.7)	49(56.3)	3.25	0.07
**≥40(Middle age)**	26(60.5)	17(39.5)		
**A person who informed them about obesity previously**				
**Health worker**	10(58.8)	7(41.2)	0.72	0.40
**Non- Health worker**	54(47.8)	59(52.2)		
**Previously tried weight reduction**				
**Yes**	45(48.4)	48(51.6)	0.09	0.76
**No**	19(51.4)	18(48.6)		
**Weight reduction method tried before**				
**Exercise-related**	13(46.4)	15(53.6)	0.06	0.80
**Others**	32(49.2)	33(50.8)		
**Family member with obesity**				
**Yes**	49(48.0)	53(52.0)	0.27	0.60
**No**	15(53.6)	13(46.4)		

About half of the participants who achieved the weight target reduction 53 (52.0%) had a family history of obesity, which was highest in the siblings 6 (64.5%).

More of those who achieved target weight reduction had low support 4 (57.1%). Table 3 shows that of the participants with minimal or mild depression, half 5 (50.0%) were able to attain target weight reduction while more participants with normal self-esteem, 65 (52.0%), achieved target weight reduction. Over half of the participants, 49 (52.7%) with a low level of concern in the EAT-26 score, achieved target weight reduction.

**Table 3:** Association between the psycho-emotional factors of participants and target weight reduction

Psycho-emotional variables	No target weight reduction n=64	Target weight reduction n=66	χ^2^	p-value
**Multidimensional Scale of Perceived Social Support** **(MSPSS)**				
**Low Support/Acuity**	3(42.9)	4(57.1)	1.13	0.57
**Moderate Support/Acuity**	25(44.6)	31(55.4)		
**High Support/Acuity**	36(53.7)	31(46.3)		
**Zung Depression Scale (ZDS)**				
**Normal**	59(49.2)	61(50.8)	0.01	0.96
**Minimal to Mild depression**	5(50.0)	5(50.0)		
** Rosenberg Self-Esteem (RSE)**				
** Low self-esteem**	4(80.0)	1(20.0)	1.97	0.16
**Normal self-esteem**	60(48.0)	65(52.0)		
**Garner's Eating Attitude Test (EAT-26)**				
**Low level of concern**	44(47.3)	49(52.7)	0.48	0.49
**High level of concern**	20(54.1)	17(45.9)		

Table 4 indicates that a greater proportion of participants with calorie loss above 500kilocalories, 16(55.2%), could achieve target weight reduction. Among those who were active, a lower proportion, 13 (43.4%), achieved target weight reduction, while among those who were inactive/ sedentarily active, a higher proportion, 53 (53.0%), achieved target weight reduction. Over half of the participants who did not consume alcohol, 59 (52.2%), reached the target weight reduction.

**Table 4:** Association between lifestyle, biochemical and anthropometric factors of participants and target weight reduction

Lifestyle variables	No target weight reduction n=64	Target weight reduction n=66	χ^2^ p-value	
***Dietary***				
***Calorie change (kilocalories):***				
***Calorie loss <500***	32(52.5)	29(47.5)	0.53	0.77
***Calorie loss ≥500***	13(44.8)	16(55.2)		
***Calorie gain***	19(47.5)	21(52.5)		
***Physical Activity***				
***Inactive***	47(47.0)	53(47.0)	0.86	0.35
***Active***	17(57.6)	13(43.4)		
***Tobacco use***				
***Yes***	3(100.0)	0(0.0)	3.17	0.12
***No***	61(48.0)	66(52.0)		
***Alcohol use***				
***Yes***	10(58.8)	7(41.2)	0.72	0.40
***No***	54(47.8)	59(52.2)		
***+WC change (cm)***				
***WC non-reduction***	23(60.5)	15(39.5)	2.74	0.10
***WC reduction***	41(44.6)	51(55.4)		
***++WHR change***				
***No WHR improvement***	39(47.6)	43(52.4)	0.25	
***WHR improvement***	25(52.1)	23(47.9)		
***Total Cholesterol (mg/dl)***				
***Normal***	39(50.7)	38(49.3)	0.15	
***Elevated***	25(47.2)	28(52.8)		
***Triglycerides (mg/dl)***				
***Normal***	48(45.7)	57(54.3)	2.7	
***Elevated***	16(64.0)	9(36.0)		
***HDL-C (mg/dl)***				
***Normal***	55(49.6)	56(50.4)	0.31	
***Low***	9(47.4)	10(52.6)		
***LDL-C (mg/dl)***				
***Normal***	21(44.7)	26(55.3)	0.61	
***Elevated***	43(51.8)	40(48.2)		

*Normal values for Fasting Lipid Profile: Total Cholesterol (TC)= 120–250 mg/dl, Low-Density Lipoproteins-Cholesterol (LDL-C) = 80–120 mg/dl, High-Density Lipoproteins- Cholesterol (HDL-C) = (35–86) > 40 mg/dl men, >50 mg/dl women and Triglycerides (TG)= 54–115 mg/dl.

+Waist Circumference (WC)

++Waist Hip Ratio (WHR) Improvement: When the WHR value de-creases. Normal: WHR < 0.95 and < 0.85 in males and females respec-tively, while > 0.95 and >0.85 is undesirable in males and females re-spectively. 40

A higher proportion of participants with elevated TC 28 (52.8%) and low HDL-C 10 (52.6%) at baseline achieved target weight reduction. Over half of those with WC reduction, 51 (55.4%) achieved the target weight reduction.

In Table 5, the statistically significant independent determinants of weight reduction were knowledge of Total Cholesterol [TC] (p=0.01) and Low-Density Lipoprotein Cholesterol [LDL-C] (p=0.03). Knowledge of HDL-C (p=0.06) and EAT-26 score (p=0.08) were also independent determinants of weight reduction which showed a tendency of significance. The proportion of variance explained by the model tested was R^2^=0.3928, with the adjusted R^2^ equal to 0.2106. The statistical model derived using the coefficients was: Weight reduction = 0.0028 (LDL-C) -0.029(TC) -0.053 (EAT-26) +0.041(HDL-C) The one-way repeated measures ANOVA (RANOVA) test carried out between the participants' baseline weight and the weight at the end of the study also showed that there was a significant effect of counselling using the PILs with the lifestyle modification mnemonic ABC, leading to mean weight reduction from 97.2±16.4kg to 95.6±14.2kg in the study participants [F-statistic (2,128), p<0.05].

**Table 5:** Multiple linear regression of the determinants of weight reduction among participants with weight reduction

	Coefficient	se	t	p-value	Lower CI	Upper CI
**Variables**						
**Obesity age**	-0.012	0.029	-0.415	0.680	-0.07	0.05
**Age**	0.006	0.039	0.168	0.867	-0.07	0.08
**Income**	0.000	0.000	-2.328	0.023	0.00	0.00
**Height 1**	-6.359	3.838	-1.657	0.103	-14.04	1.32
**WC 1**	0.006	0.037	0.159	0.874	-0.07	0.08
**WHR1**	0.122	4.306	0.028	0.978	-8.49	8.73
**BP Diastolic 1**	0.015	0.023	0.631	0.530	-0.03	0.06
**BP Systolic 1**	0.007	0.034	0.195	0.846	-0.06	0.08
**HDL-C**	0.041	0.021	1.929	*0.059	0.00	0.08
**LDL-C**	0.028	0.012	2.217	**0.030	0.00	0.05
**MSPPS total 1**	-0.095	0.269	-0.355	0.724	-0.63	0.44
**RSE score baseline**	-0.121	0.086	-1.413	0.163	-0.29	0.05
**EAT-26 score 1**	-0.053	0.030	-1.763	*0.083	-0.11	0.01
**IPAQ Total 1**	0.000	0.000	-0.315	0.754	0.00	0.00
**Triglycerides**	0.015	0.010	1.566	0.123	0.00	0.03
**Total Cholesterol**	-0.029	0.011	-2.675	**0.010	-0.05	-0.01
**ZDS score**	-0.058	0.043	-1.329	0.189	-0.14	0.03
**Calories 1**	0.001	0.000	1.419	0.161	0.00	0.00
**Constant**	15.314	8.275	1.850	0.069	-1.24	31.87

p* <0.1 P** <0.05 Prob > F = 0.0139 R-squared = 0.3928 Adj R-squared = 0.2106

## Discussion

This study showed that 50 per cent of the participants were able to meet the recommended weight reduction target, with the majority seen in the participants less than 40 years, and this was corroborated by the report by Atlee et al., in 2017.^41^ There was a female preponderance observed among those who were able to achieve target weight reduction similar to a study by Andreyeva et al., 42 with most weight reduction in unmarried participants which was contradictory to an exploratory study with a cross-sectional study design, in which more married participants reduced in weight.^41^ Those participants who had greater than or equal secondary school education in this study reached the target for weight reduction. This observation was not supported by the report by Barbering et al., in 2018, in which it was found that education was not related to trying to reduce weight.^43^ Majority of those with blue-collar jobs achieved target weight reduction, which differed from a report in which weight reduction was higher in public sector workers. 44

Those who had an income less than 18,000 nairas ($ 51.4-the minimum standard wage as of the period of this study) were able to achieve target weight reduction from an article that individuals with less income could not access weight control methods as well as others.^45^

Most of the women who achieved target weight were above 45 years, and the majority of the participants who were able to achieve target weight reduction had a positive family history of obesity in mostly siblings, which was corroborated by a study in which there was a greater weight loss in those with a positive history. 46 A few participants reached the target weight and were informed by health workers, corroborating a report.^42^

In this study, those who achieved the target weight reduction had low support. This contradicts the study in which social support groups give improved weight outcomes. 47 Half of the respondents with minimal or mild depression were able to achieve target weight reduction, which was supported by authors who suggested an advantage in treating depression, especially in those who were single.^48^ More respondents with normal self-esteem reached the target weight reduction, which agreed with the study in which high levels of confidence and self-esteem affected weight loss. 47 There was a lower level of concern observed in participants with target weight reduction from the EAT-26, supported by reports that documented that one of the barriers to weight reduction was abnormal eating, such as binge eating. 49

Many participants who lost above 500 kilocalories could have more target weight reduction than those who lost less than 500 kilocalories.

The mean change in calorie intake was -257.4 kilocalories. Therefore, a reduction in calorie intake was achieved and was supported by a document.^50^ Interestingly, in this study, both calorie reducers and calorie gainers were found to achieve weight reduction, which was corroborated by authors who opined that with weight reduction came a reduction in Leptin level and invariably a positive energy balance. Thus, food intake could exceed energy expenditure in those trying to reduce weight.^14^ Most of the participants with obesity (88.1%) were documented to ingest a high-calorie diet (p>0.05) in the hospital-based study in South Western Nigeria. 38 Calorie loss, on its own, may not lead to weight reduction and should be combined with exercise as it has been documented that the body will compensate once there is calorie reduction and cause physical activity to decline.^51^

Though some participants who reduced in target weight at the end of the study were observed to be more physically active, even in those who were inactive or sedentary, a larger proportion reduced in target weight. A report supported this finding of weight reduction with increased activity.43 In contrast, these bidirectional findings between physical activity, sedentary activity, and obesity outcomes were corroborated by documentation of an inverse association between sedentary activity and abdominal obesity in females. 36 More respondents who did not consume alcohol and participants who did not take cigarettes were able to reach the target weight reduction, which was not in sync with previous reports.^52^,^53^ WC helps in predicting health outcomes in both men and women 54, and more respondents were able to achieve target weight reduction with WC reduction. More of the participants with dyslipidemia, which aligned with a report which documented that dyslipidemia in participants with obesity was 40.7%^38^ were able to reduce in target weight.

Following multiple linear regression analyses, the statistically significant predictors were TC (p=0.01) and LDL-C (p=0.03). HDL-C (p=0.06) and EAT-26 score (p=0.08) were independent determinants of weight reduction which showed a potential for significance.

Counselling with PILs incorporating the lifestyle mnemonic ABC demonstrated a significant effect on the mean weight reduction from the first visit to the last visit.

## Conclusion

Counselling with Patient Information Leaflets on the ABC mnemonic for lifestyle modification is effective in weight reduction. The public health implication of this study is that weight reduction could be achieved by enhancing eating attitudes and disclosing the fasting lipid profile of the participants.

A statistical model based on the Fasting Lipid Profile for predicting weight reduction among patients with obesity was successfully developed and could be applied in managing obesity among patients. Future studies may include the measurement of Leptin levels in participants.
